# Investigating Primary School Nurses' Activities That Are Effective in Health Promotion and Primary Prevention: A Systematic Review

**DOI:** 10.1111/josh.70027

**Published:** 2025-06-25

**Authors:** Lisa Kühne, Faith Mugo

**Affiliations:** ^1^ SOCIUM Research Center on Inequality and Social Policy University of Bremen Bremen Germany

**Keywords:** health promotion, primary prevention, primary school students, school nurse, systematic review

## Abstract

**Background:**

School nurses that are regularly present at school can influence health behaviors in the early lifespan, establishing a foundation for healthy growth. This systematic review identifies activities targeting health promotion and primary prevention and assesses the activities that are effective in improving students' health outcomes.

**Methods:**

The protocol was registered on PROSPERO (CRD42023471364). The search was conducted in the databases MEDLINE, PsycINFO, CINAHL, ERIC, Scopus, and ASSIA.

**Findings:**

The 19 identified studies include seven RCTs, four quasi‐experimental studies, four cross‐sectional studies, one secondary data analysis, one qualitative study, and two reviews published between 2012 and 2023. The activities were categorized as health education, screening, and structural prevention. Certain health education activities, commonly in short sessions and incorporating interactive and creative features, effectively improved health behavior, knowledge, and physical health outcomes. One screening intervention reliably identified children with vision abnormalities at an early stage.

**Implications for School Health Policy, Practice, and Equity:**

By implementing these activities, school nurses enhance students' health behaviors of nutrition, physical activity, toothbrushing, and nail‐biting, among others. In this way, they promote children's well‐being and are well positioned to reduce health inequalities.

**Conclusions:**

Primary schools are an ideal setting to engage students and their families in enhancing their health literacy, with school nurses incorporating significant potential for public health.

## 1 Introduction

Health behaviors are established in early childhood and influenced by family and community background, including the school environment. The primary schools serve as a relevant setting for the provision of social, educational and health guidance in the early lifespan to ensure the baseline for growing up healthy [1, 2]. Students in primary schools are easy to reach in this setting as they inherently attend school every day for several years and are influenced not only by education in class but also by the social surrounding. Moreover, within schools, various groups of students with different socioeconomic backgrounds may be reached [1].

Schools as a health promoting setting foster education and health in their context and provide a healthy environment with an outreach to the community. In this way, students, families, teaching and other school staff, as well as external health providers are engaged in efforts to reach their potential in supporting children. These promoting school health services include health education, counseling, and social support with a focus on the topics of nutrition, physical activity, and recreation, and mental health [3].

This promising approach to health promotion and primary prevention as well as health literacy enhancement can be implemented by qualified school staff, particularly by school nurses. School nurses working in public and private schools typically serve as the first contact for medical care, conduct surveillance, and provide primary prevention and health promotion [4, 5]. Based on their professional nursing skills and local network, and being regularly present at the school, school nurses can provide individualized and population‐based health literacy promotion [5, 6]. This complex intervention includes various activities aiming for multiple health, academic, and social outcomes in a heterogeneous target group with different social backgrounds. In this way, school nurses may contribute to the bridging of health inequality [1].

To date, findings on concrete activities that improve health outcomes are limited. Some reviews previously reported on the effectiveness of school nurses' interventions [1, 4, 5, 7, 8, 9, 10]. Mostly, the reviews reported on activities of direct care, chronic disease management, counseling, surveillance, and health promotion in different school settings. Effective health promotion and prevention measures include, for example, simple health education lessons, cultural and gender‐sensitive physical activity programs, and screening for obesity, diabetes mellitus, bronchial asthma, or hearing impairment [5]. Whereas on the structural prevention level, for example, assisting teachers with the integration of healthy messages into the school curriculum [5] or collaboration with food service staff, physical education staff, and community health professionals [2] have been established. Previous studies generally considered health promotion and primary prevention rather as an additional order besides direct care.

To assess the extent of school nurses' activities targeting health promotion and primary prevention, this systematic review aimed to answer the following review questions:

– Which are primary school nurses' activities to deliver health promotion and primary prevention?
– Which activities are effective in improving health outcomes in children?

The objective of this systematic review was to identify activities with a focus on the practical implementation in the primary school setting.

## 2 Methods

This review was conducted according to the Cochrane guideline for systematic reviews of interventions [11]. The protocol was published on PROSPERO under CRD42023471364 [12]. The search strategy was created with consultation of a librarian experienced in public health research and finalized after pilot searches. The relevant databases that have been searched are MEDLINE (via Ovid), PsycINFO (via Ovid), CINAHL (via EBSCO), Education Resources Information Center (via EBSCO), Scopus, Applied Social Sciences Index, and Abstracts (via ProQuest). The search was conducted on 12.09.2023 and limited to references published after 2012 to restrict the results to current findings from the last decade. The syntax combined the terms “primary school,” and “school nurse,” and “health promotion,” or “prevention,” in adaptation to the targeted databases (see Appendix C).

Publications in indexed and peer‐reviewed journals as well as unpublished and gray literature were eligible. Studies published in both English and German language were considered for inclusion. This review comprises various study designs, namely experimental and quasi‐experimental studies, descriptive and analytical cross‐sectional studies, and reviews that process primary data. Inclusion and exclusion criteria were defined following the PICO statement (population, intervention, comparison, outcome). The *population* are primary school students (aged 6–11) receiving a health promotion intervention either delivered or supported by the school nurse. Acutely and severely ill children were excluded since the activities of medical care are not relevant to the research questions. The *interventions* comprised any activity of health promotion or primary prevention provided by the school nurse. Studies on school nurses exclusively delivering medical care were excluded. Whenever multiple interventions were reported, studies were included if the activities can be distinguished to solely include those for health promotion or primary prevention. Studies with no *comparator* or any control group were included, for example, before and after design or waiting list. The *outcomes* comprised health outcomes referring to physical and mental health measures; outcomes of health behavior, for example, quantity and intensity of physical activity, or health literacy; and outcomes of primary prevention, for example, vaccination rate. Studies solely reporting academic outcomes were excluded.

**FIGURE 1:**
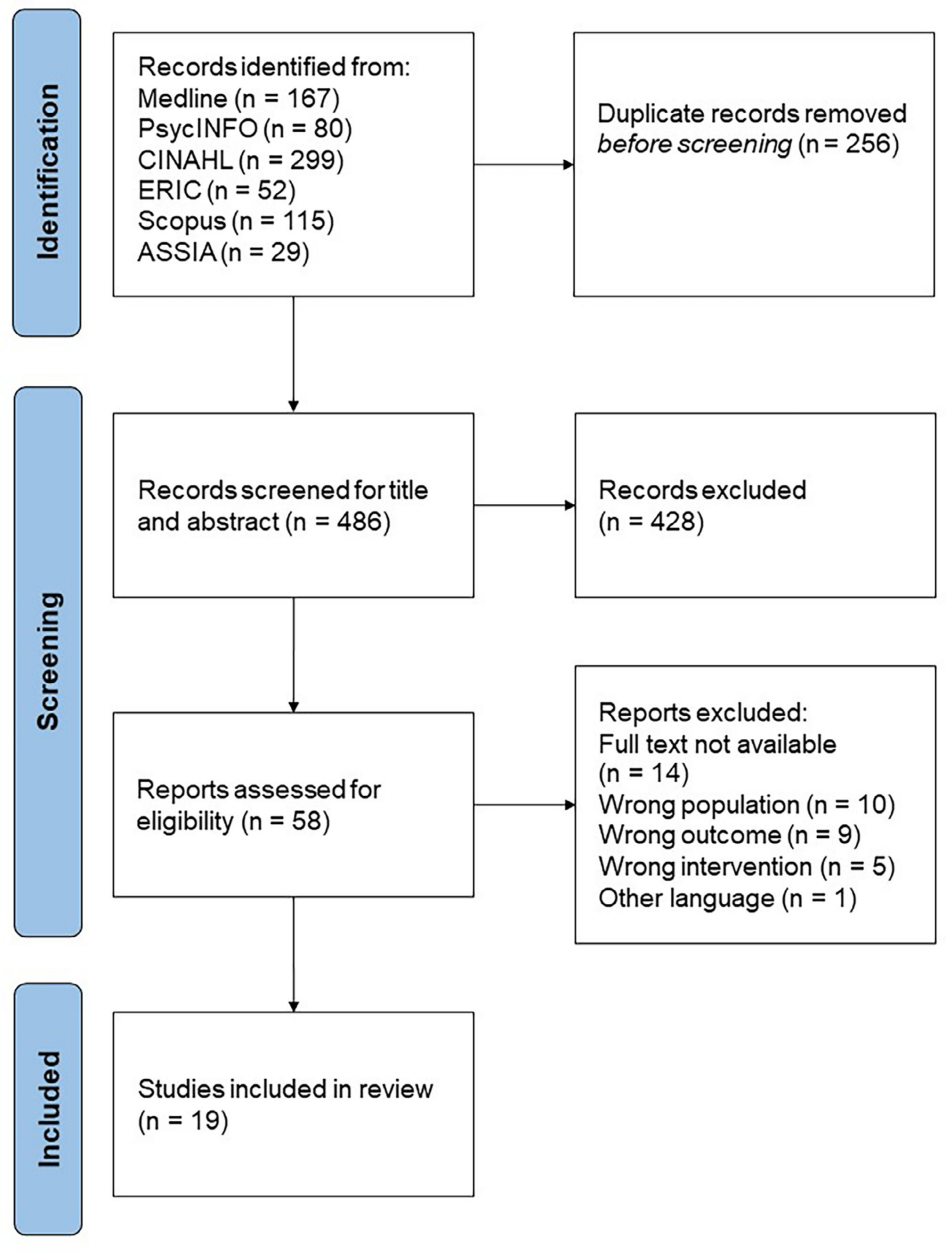
PRISMA flow diagram of the included studies.

The records identified from the databases were reduced by the duplicates. Titles and abstracts were then screened by two reviewers (L.K. and F.M.) independently for assessment against the inclusion criteria. The full texts of the selected studies were then assessed in detail by L.K. and F.M. independently, and records not meeting the inclusion criteria were excluded. Any disagreements that arose between the reviewers at each stage of the selection process were resolved through discussion. The results of the study selection process are presented in a Preferred Reporting Items for Systematic Reviews (PRISMA 2020) flow diagram [13] (see Figure 1). Potential risk of bias was assessed using validated tools suitable to each study design, namely RoB 2 for randomized trials [14], ROBINS‐I for non‐randomized studies of interventions [15], AMSTAR 2 [16] for the systematic review, JBI critical appraisal checklist for qualitative research [17], JBI critical appraisal checklist for textual evidence in narrative reviews [18]. One reviewer (L.K.) applied the assessment tools, and the other reviewer (F.M.) controlled the appraisal.

Bibliographic information and details on the study design, participants, type of activity, and health outcomes were gathered by one reviewer (L.K.) and checked by another reviewer (F.M.). The activities of primary school nurses were described and analyzed in alliance with the MRC guideline for evaluating complex interventions [19]. Therefore, the interventions were evaluated with regard to the degree of implementation and the context, referring to the practical setting and the corresponding health and school system. Specifically, the targeted populations were outlined under a diversity perspective with a focus on if and how the social background of the families is addressed. The effective activities were analyzed according to the primary school students' health outcomes.

## 3 Findings

The presented search revealed 19 studies for inclusion. The number of studies in each stage of the selection process can be traced in the PRISMA flow diagram (see Figure 1).

This review includes seven RCTs, four quasi‐experimental studies, four cross‐sectional studies, one secondary data analysis, one qualitative study, one narrative review, and one systematic review (see Table 1). Ten out of the 19 studies applied a pre‐ and post‐intervention measurement. The presented activities aimed at promoting health and/or preventing conditions such as obesity [27] or at early detection of, for example, bronchial asthma [36]. The studies incorporated at least some risk of bias due to the setting (see Appendix B for detailed information on potential bias).

**TABLE 1:** Overview of studies reporting activities of health promotion and primary prevention.

Authors, year, country of publication	Study design	Aim of the intervention	SN activity	Outcome	Risk of bias
*Studies reporting activities on health education* (*n* = *13*)					
Güler et al. 2023, Turkey [20]	RCT	General health promotion	12 weekly interactive online sessions of 40 min after school hours^a^	Improved self‐reported food behavior, physical activity self‐efficacy, and health perceptions and behaviors	Moderate
Kim et al. 2016, South Korea [21]	RCT	General health promotion	10 weekly interactive sessions of 40 min with creative features like role‐play, dancing, painting^a^, ^b^	Improved self‐reported behaviors of disease prevention and safety, but not statistically significant for health promotion	Serious
Goodarzi et al. 2020, Iran [22]	RCT	Oral health promotion	4 weekly interactive sessions of 50 min + students trained as ambassadors to promote oral health in class every 2 days for another 6 months^a^	Improved self‐reported oral health behavior, perceived benefits, and self‐efficacy; less dental plaque assessed by dentist	High
Çövener‐Özcelik et al. 2017, Turkey [23]	RCT	Prevention of fecal‐orally transmitted diseases	Single 40 min interactive session + brochures, banners in school restroom^a^	Improved self‐reported hygiene habits and knowledge immediately after the intervention, but not 3 months later	High
Ergun et al. 2013, Turkey [24]	RCT	Stop nail‐biting behavior	Individual plan on nail‐growing goal and follow‐up for 8 weeks + single presentations of 35 min to students in groups and to families in groups^a^	Less nail‐biting assessed by SN during follow‐up	Some concerns
Ergün et al. 2012, Turkey [25]	RCT	Prevention of school injuries	2 interactive sessions of 40 min + activity leaflet by SN to prepare for child‐to‐child training; these children provided training to other children 1 week later^a^	Child‐to‐child training improves students' attitudes towards security measures as effectively as instructor‐to‐child training	High
Szilagyi et al. 2019, United States [26]	RCT	Influenza vaccination compliance	3 text message reminders to parents^b^	No difference in vaccination rates compared to usual vaccination reminders	Some concerns
Lovell 2018, United States [27]	Quasi‐experimental study	Obesity prevention	Team challenge on daily health behavior (nutrition and physical activity) for 2 weeks^b^	Points gained for healthy behavior, e.g., fruit and vegetable intake; chocolate milk consumption decreased during the intervention, but increased afterwards	Serious
Tucker et al. 2015, United States [28]	Quasi‐experimental study	Obesity prevention	4 sessions of 10–15 min + weekly individual follow‐up for 7 months (School A) and 3 months (School B)^a^	Increased number of steps measured by pedometer; increased self‐reported fruit and vegetables intake; no effect on BMI as measured by SN	Serious
Gür et al. 2017, Turkey [29]	Quasi‐experimental study	Stop nail‐biting behavior	Weekly individual follow‐up + 4 group sessions of 30 min + 4 family group sessions of 40 min^a^	Less self‐reported nail‐biting and as assessed by SN	Serious
Henry et al. 2022, United States [30]	Quasi‐experimental study	Mindfulness	12 sessions in 8 weeks + rewards for students^b^	Improved self‐reported mental health (not statistically significant)	Serious
Mbakaya and Lee 2019, Australia [31]	Qualitative study	Hand hygiene	Daily to weekly sessions for 6 months + briefing of school personnel and parents + take‐home posters and leaflets	Feasibility and acceptability for children, parents and school personnel; improved hand hygiene practices	Some concerns
Lineberry and Ickes 2015, United States [8]	Systematic review	General health promotion, hand hygiene, and obesity prevention	Education sessions to students and school personnel in different frequencies and durations	Improved self‐reported nutrition behavior; increased knowledge on nutrition, physical activity, and mental health; increased vaccination rates and reduced infection transmissions in schools	Critically low confidence in the results of the review
*Studies reporting activities on screening* (*n* = *6*)					
Li et al. 2022, Australia [32]	Cross‐sectional study	Prevention of visual loss	Dual‐modality vision screening once^a^	Reliable detection of vision abnormalities through combined tests	Moderate
Cheng et al. 2021, China [33]	Cross‐sectional study	Strabismus detection	3 photos taken with app “EyeTurn” on SN's smartphone once	Reliable detection of ocular misalignment, further in‐person testing	Moderate
Moron 2023, United States [34]	Cross‐sectional study (for screening results)	Oral health promotion in underserved areas	Screening for caries and fluoride varnish application once + oral health education + oral hygiene products	Reliable identification of students in need and transfer to early dental care; high prevalence of untreated caries	Critical
Anderson et al. 2016, New Zealand [35]	Secondary data analysis (for screening results)	Prevention of rheumatic fever	Throat swabbing for Streptococcus once + daily individual follow‐up	Detection of early‐stage *Streptococcus* infection	Low
Rabner et al. 2020, United States [36]	Cross‐sectional study	Bronchial asthma detection	Four self‐report questions once in combination with SN records and school absenteeism data	Appropriate identification of students with bronchial asthma	Serious
Lineberry and Ickes 2015, United States [8]	Systematic review	Detection of visual loss, obesity prevention, vaccination compliance	Vision screening, BMI screening, tracking of vaccinations	Early detection of abnormalities, followed by individual consult; healthy weight program in affected schools	Critically low confidence in the results of the review
*Structural prevention* (*n* = *2*)					
Mbakaya and Lee 2019, Australia [31]	Qualitative study	Hand hygiene	Proper hand hygiene facilities, posters with instructions	Feasibility and acceptability for children, parents, and school personnel; improved hand hygiene practices	Some concerns
Tipton 2015, United States [37]	Narrative review	Reduced sugar‐sweetened beverage intake	Banning the sale of sugar‐sweetened beverages in school, healthy alternatives, limiting portion sizes in mensa	Well‐positioned role of SN to promote more healthful beverages	Low

Abbreviations: RCT, randomized clinical trial; SN, school nurse.

^a^ SN activity evaluated as effective in improving the health outcome.

^b^ SN activity evaluated as not effective in improving the health outcome.

The reviewed studies did not involve potential users in intervention development given the difficulty to consult children of that age group. Some (6/19) incorporated prior feedback from school personnel and experts [20, 21, 23, 26, 30, 37] and the interventions in 3/19 studies were following a pilot study [28, 32, 35]. The other reported activities were implemented with a theoretical background based on literature review, such as the oral health education reinforced by the peer group that relies on the Health Belief Model [22] or the hand hygiene program that comprises the WHO's Health Promoting Schools framework [31].

### 3.1 Health Education

Most of the identified activities, in 13/19 studies, can be categorized as health education sessions. The topics contain general health promotion [8, 20, 21] with contents on nutrition, physical activity, personal hygiene, mitigating peer bullying, and first aid in different sessions. Other activities focused on specific topics like stopping nail‐biting [24, 29], toilet hygiene and hand washing [23, 31], obesity prevention [27, 28], school injury prevention [25], mindfulness [30], and influenza vaccination [26]. The school nurses conducted sessions mostly with students in groups in their regular classroom, while one program was held online due to the pandemic [20]. The contents were practically delivered using slideshow presentations [21, 25] and trained in an interactive and creative way [21, 22, 23, 24, 27], for example, with guided group work [22] and discussions [20]. Two health education programs applied child‐to‐child training [22, 25] while another school nurse activity was accompanied by peer group reinforcement and repetition [22, 26]. School nurses hung posters and leaflets [26, 31] and some added health education sessions also to families in groups encouraging them to support the students [24, 28, 29]. For other activities, school nurses sent reminder text messages for the upcoming influenza vaccination [26] or asked parents individually to reinforce students' learnings at home [22, 31].

### 3.2 Screening

Six out of 19 studies reported on school nurse activities that incorporate some type of screening. The school nurses reviewed students' eyes for vision abnormalities and assigned students with a conspicuous result for further assessment by an eye health professional [8, 32, 33]. Others conducted screening for BMI [8] or for oral health [34], where the school nurses identified students with caries and gave their consult for oral hygiene products and, potentially, further care. In communities with at‐risk populations for infectious diseases, school nurses did throat swabbing for a test for Streptococcus once and assessed students identified as positive daily and treated sore throats and skin infections [35]. Another screening activity was conducted in an area with high bronchial asthma prevalence. The school nurses obtained students' questionnaires and combined the results with their own records and school absenteeism data to triage students and coordinate medical care if necessary [36].

### 3.3 Structural Prevention

Two out of 19 studies reported on school nurses' activities in structural prevention. Besides the health education sessions on hand hygiene for students, the school nurses had proper handwashing facilities installed and hang posters reminding to wash hands with the correct technique [31]. To reduce the intake of sugar‐sweetened beverages, school nurses set up a healthy school environment, for example, by banning the sale of sugar‐sweetened beverages and offering water and other alternatives and limiting portion sizes in the school mensa. Moreover, they support national policies such as prohibiting the marketing of sugar‐sweetened beverages to students and work toward establishing wellness councils in the school or the community [37].

### 3.4 Effective Primary School Nurses' Activities

Most of the studies (12/19) made a statement on the effectiveness of the presented school nurses' activities comparing the outcomes in the intervention group either pre and post intervention or to a control group in another school or class receiving no intervention or receiving the intervention after evaluation. The outcomes were self‐reported [20, 21, 22, 23, 25, 28, 30], assessed by a pedometer [28], assessed by the school nurse [24, 29] or other professionals [22, 32] during follow‐up or drawn on an environmental level, namely as the vaccination rate [26] or orders of chocolate milk as reported by food service personnel [27].

Of these, nine studies concluded that the intervention improved health outcomes with statistical significance [20, 21, 22, 23, 24, 25, 28, 29, 32].

The effective health education sessions improved students' health behavior, namely toward nutrition and physical activity [20, 28], oral health [22], hygiene [21, 23], reducing nail‐biting [24, 29] and injury prevention [25]. These sessions were mainly conducted weekly and for a longer term of several months. Three health education activities were accompanied by individual follow‐up [24, 28, 29]. Generally, health education was partly reinforced by peer students [22, 31]. Two studies also presented child‐to‐child training that was instructed by the school nurses [22, 25]. Some activities included either teachers and other school personnel [22, 27, 31, 36] and/or the students' families [22, 29, 31], for example, with take‐home health education information. Some health education activities incorporated interactive features [20, 22, 23], creative sessions [21, 24], and some other form of gamification, such as a quiz, skits, a song competition [37], or a team challenge [27]. The interventions partly included some kind of reward, that is either related to the health aim, such as nail clippers and files [24] or a pedometer, frisbee, and water bottle [28]. Other rewards were of a social nature, such as a family celebration meal [28]. Some school nurses honored completion of the program with a certificate [27, 28] and a gift card [30], stickers [24], or a bookmark [27].

One screening activity reliably detected students with reduced vision acuity or abnormalities to assign them to further assessment by an eye health professional. The school nurses conducted a photoscreener and a visual acuity test in one examination. In comparison to assessments by external eye health professionals, the school nurses' test showed a reliable positive predictive value of 0.91 [32].

Another three studies made a statement on effectiveness but found no beneficial effect on students' health [26, 27, 30]. The three text message reminders sent to parents in addition to the influenza vaccination program with backpack papers and autodialed phone reminders did not increase the vaccination rates compared to the control group that only received the regular information [26]. The activity and nutrition team challenge led to decreased orders of chocolate milk as reported by food service personnel, but this trend was not continued 1 month afterwards [27]. The mindfulness education sessions delivered by the school nurse while the regular teacher was present in the classroom improved students' self‐reported mental health immediately after the intervention, but the effect was not statistically significant [30].

## 4 Discussion

This systematic review identified primary school nurses' activities that first of all target primary students' health promotion and primary prevention. Most of the activities were in the form of health education, commonly in short sessions and incorporating interactive and creative features. Besides health education, school nurses conducted screening, for example, for vision and oral health as their contribution to primary prevention. Other activities addressed the structural conditions in the primary school.

The identified activities targeted students' health behavior, including physical activity, nutrition, hygiene, mindfulness, and stopping nail‐biting. Primary prevention interventions aimed to increase vaccination rates among students and to allow for an early detection of streptococcus infection, vision abnormalities, caries, bronchial asthma, and obesity. The education sessions addressed health promotion in general [20, 21, 28] or concrete topics such as nail‐biting behavior [24, 29]. Some interventions of general health education incorporated physical activity promotion among other topics [20, 21, 27, 28], but no study targeted exclusively students' physical activity. Only one study was exclusively on promoting mental health [30]. Other studies incorporated mental health topics within the general health education sessions [20, 21]. Similarly, another literature review on health promotion in primary schools identified solely a few interventions addressing mental health [1]. While proper mental health is a critical resource at a young age, evidence indicates that young students already experience mental health challenges [3]. These needs are addressed in secondary schools, for example, school nurses are capable of promoting adolescents' mental health with healthy behaviors of how to prevent or suitably deal with stress [38]. These learnings from secondary schools imply that education and intervention should begin earlier, at primary school age, to build a foundation for resilience and overall well‐being as individuals grow.

Most of the studies made a statement on the effectiveness of the presented activities on health outcomes, but the evidence is limited according to the quality level of data collection. Generally, RCTs on interventions are more reliable, but for this context also the included quasi‐experimental studies gained importance. Mostly, these studies generated results out of measurements within the same group pre and post intervention. Even among the included RCTs, the risk of bias appeared to be at least moderate. Given the setting of applied activities that cannot be blinded to the observed probands, the presented results are interpreted with appropriate caution. Generally, knowledge on effective school nurses' interventions is limited due to the methodological difficulties and the resulting lack of quantitative and robust data [5].

Nevertheless, the identified effective activities of health education share characteristics like rather short sessions, repeated for several months, as stated similarly for health promotion interventions by regular school staff [1]. Also, these school interventions included some form of individual follow‐up with the school nurse [1]. Effective interventions identified in this review also partly engage peer students to take over this role. Other similar health education sessions by school nurses in primary, middle, and high schools were found to be effective in enhancing health behavior and health literacy [5]. These previous reviews also point out that the interventions should involve the school personnel and the students' families [1, 5], which also applies to the effective activities identified in this review. For the specific target group of primary school students, the social aspect is crucial, and incorporating interactive features and offering rewards enhance motivation.

The findings are limited by the heterogeneity of the school nurses' activities. The primary studies reported on interventions differently, partly not in much detail, especially not on the social background of the target group. Regarding the evaluation of these complex interventions [19], the primary studies were analyzed with a focus on the practical implementation and the context of the activities, including the role of the school nurse in the corresponding health and school system. Further research that adds to the evidence of feasible interventions should consider the adaptation in the school setting, practically how to implement the activity in the school day aligning with regular health services. Identifying school nurses' best practices is crucial for successful integration.

### 4.1 Implications for School Health Policy, Practice, and Equity

School nurses showed their potential to enhance students' health unprecedentedly during the COVID‐19 pandemic. During this time, school nurses adopted suitable hygiene routines, briefed students and school personnel on adhering to an established concept, and contributed to immunization within the community [5]. Moreover, school nurses were well‐positioned to speak to students regarding their concerns about the infection itself, as well as their academic performance and social isolation. These circumstances impacted children's mental health which school nurses can address to prevent long‐term negative consequences [39].

Persistently, stakeholders should acknowledge and strengthen the school nurses' work addressing major health concerns like unhealthy nutrition and physical activity, lack of hygiene, poor oral health, communicable diseases, injuries, and mental health challenges [3]. Besides medical care, activities of health promotion and primary prevention are highly recommended to be implemented in the regular school curriculum. Conducting effective health education, school nurses are well‐positioned to enhance health literacy during this period of life [6]. Therefore, they should offer rather short sessions given the young students' ability to concentrate. To repeat and deepen learning, the health education intervention should run for a longer period of several months accompanied by follow‐up, when suitable. Students can be attracted by active features and their engagement can be honored by some form of reward.

Besides health education, school nurses can conduct screening themselves or escort students to external healthcare providers within the community or support families with additional information. With extensive knowledge of the healthcare system, they can advise families on their rights and entitlements. Being present at school, school nurses are well‐positioned to serve as advocates for students' health [8] and build up a health‐promoting environment. For this purpose, they should cooperate with the school personnel and other providers of health and social services in the community. School nurses can add to the knowledge, and vice versa, by collaborating with other professionals.

Primary school nurses should involve the families to address the social background in this target group of young children. Specifically, school nurses should be sensitive to the social determinants of health and outline the target group from a diversity perspective. By taking into account families' economic and cultural resources, especially when addressing more deprived children and families effectively, school nurses are well‐positioned to bridge health inequality and social inequality in the long term [40].

This review's findings underscore the relevance of primary school nurses for the public interest. To adhere to their potential to support children in growing up healthy, school nurses need reliable resources and support from public stakeholders, school administrations, and families [3].

## 5 Conclusion

This systematic review identified activities of health promotion and primary prevention conducted by school nurses in primary schools, categorized as health education, screening, and structural prevention. Health education activities and screening for vision abnormalities can improve students' health. School nurses are well‐positioned to implement these activities in the primary school setting and contribute to the bridging of health inequality in communities.

## Ethics Statement

Preparation of this paper did not involve primary research or data collection involving human subjects, and therefore, no institutional review board examination or approval was required.

## Conflicts of Interest

The authors declare no conflicts of interest.

## Data Availability

The data that supports the findings of this study are available in Supporting Information of this article.

## Appendix A See Table A1

**TABLE A1:** List of studies for full‐text screening.

Authors	Year	Title	Journal	Volume	Issue	Page	DOI
	2014	Inter‐Professional Collaboration Between Dental Hygiene and Elementary School Health Nurses in One North Carolina County	*Journal of Dental Hygiene*	88	5	328–328	
L. Abrantes Nicácio; R. M. Barbosa Davim; M. Barbosa Oliveira; J. C. de Farias Camboim; H. R. Leopoldino Medeiros; S. Ximenes Oliveira	2017	Educational Intervention on *Aedes aegypti* Mosquito in Schools: Possibility for Nursing in the School Context	*Journal of Nursing UFPE/Revista de Enfermagem UFPE*	11	10	9771–9777	10.5205/reuol.12834‐30,982‐1‐SM.1110201710
D. Acil; S. Ayaz	2015	Screening of Visually Impaired Children for Health Problems	*Asian Nursing Research*	9	4	285–290	10.1016/j.anr.2015.06.004
S. M. Ackers; A. M. Colbert; H. E. Fraley; J. B. Schreiber	2022	Exploring Screening Practices for Child Sexual Abuse in School Settings: An Integrative Review	*The Journal of School Nursing*			105984052 21112662	10.1177/10598405 221112662
P. Anderson; J. King; M. Moss; P. Light; T. McKee; E. Farrell; J. Stewart; D. Lennon	2016	Nurse‐Led School‐Based Clinics for Rheumatic Fever Prevention and Skin Infection Management: Evaluation of Mana Kidz Programme in Counties Manukau	*The New Zealand Medical Journal*	129	1428	37–46	
R. M. Bains; A. F. Diallo	2016	Mental Health Services in School‐Based Health Centers	*Journal of School Nursing*	32	1	8–19	10.1177/10598405 15590607
L.‐M. Bergvoll; S. S. Fjelldal; A. Clancy; M. Martinussen; H. Laholt	2023	How do Public Health Nurses in Norwegian School Health Services Support Siblings of Children With Complex Care Needs?	*Scandinavian Journal of Caring Sciences*				10.1111/scs.13184
S. A. Blessing; L. L. Mendonca	2019	The Healthy Menu Project: Role of the School Nurse: Youth‐Adult Shared Partnerships Help Change a School Lunch Menu	NASN School Nurse	34	6	335–339	10.1177/1942602X 19852583
N. Boyle; J. Anderson	2014	Evaluating a Weight Management Package for Overweight Primary School Children	*British Journal of School Nursing*	9	7	339–344	10.12968/bjsn.2014.9.7.339
H. Cheng; C. George; M. Dunham; L. Whitehead; E. Denney‐Wilson	2021	Nurse‐Led Interventions in the Prevention and Treatment of Overweight and Obesity in Infants, Children and Adolescents: A Scoping Review	*International Journal of Nursing Studies*	121		104008	10.1016/j.ijnurstu.2021.104008
W. Cheng; M. H. Lynn; S. Pundlik; C. Almeida; G. Luo; K. Houston	2021	A Smartphone Ocular Alignment Measurement App in School Screening for Strabismus	*BMC Ophthalmology*	21	1	150	10.1186/s12886‐021‐01902‐w
T.‐L. Cloete; W. J. Wilson; L. Petersen; H. Kathard	2015	Identifying a Context‐Effective School Hearing Screening Test: An Emic/Etic Framework	*International Journal of Audiology*	54	9	605–612	10.3109/14992027.2015.1014575
Ç. Çövener‐Özçelik; E. Aktaş; H. Sefer; A. F. Ocakçi	2017	Impact of Toilet Hygiene Training Program on School‐Age Children	*Turkish Journal of Research and Development in Nursing/Hemşirelikte Araştırma Geliştirme Dergisi*	19	2	25–40	
C. Cox	2018	Edutainment: A Creative Solution for School‐Based Preventive Screening Orientation in Missouri	*Creative Nurs*ing	24	4	215–219	10.1891/1078‐4535.24.4.215
K. L. Drake; C. E. Stewart; M. A. Muggeo; G. S. Ginsburg	2015	Enhancing the Capacity of School Nurses to Reduce Excessive Anxiety in Children: Development of the CALM Intervention	*Journal of Child and Adolescent Psychiatric Nursing*	28	3	121–130	10.1111/jcap.12115
A. Ergun; R. Toprak; F. N. Sisman	2013	Impact of a Healthy Nails Program on Nail‐Biting in Turkish Schoolchildren: A Controlled Pretest–Posttest Study	*Journal of School Nursing*	29	6	416–424	10.1177/1059840513 481386
S. Ergün; A. Kalkim; E. Dolgun	2013	Child‐to‐Child Training for Prevention of School Injuries in Odemis, Turkey	*Journal of School Nursing*	29	5	337–342	10.1177/105984051 2471397
T. Garretty	2017	Final Visual Outcomes and Treatment Received for Children Referred from a UK Primary School Visual Screening Program: A Comparison of An Orthoptic‐Led Program With Orthoptic‐Delivered Services	*Strabismus*	25	4	184–190	10.1080/09273972.201 7.1392988
A. Goodarzi; A. Heidarnia; S. S. Tavafian; M. Eslami	2020	The Role of Repetition And Reinforcement (by peer group) in Oral Health Education Program as a Base for Health Belief Model (hbm) Among Iranian Primary Schools Students (Cluster Randomized Controlled Trial)	*Health Education and Health Promotion*	8	2	57–66	
S. Gourlay; K. Stoddart; A. Blackadder; S. Hinton; S. Penman; P. Young; M. Osborne; S. Robertson; D. Robertson; C. Robertson	2016	School‐Based Influenza Immunisation Programme Pilot: Using an Out‐of‐Hours Model	*British Journal of School Nursing*	11	1	17–22	10.12968/bjsn.2016.11.1.17
S. Gray; D. Lennon; P. Anderson; J. Stewart; E. Farrell	2013	Nurse‐Led School‐Based Clinics for Skin Infections and Rheumatic Fever Prevention: Results From a Pilot Study in South Auckland	*The New Zealand Medical Journal*	126	1373	53–61	
R. Güler; G. Kublay; H. Fırat Kılıç	2023	The Effectiveness Of A Nurse‐LED Health Development Programme on Fourth‐Grade School‐Age Children In North Cyprus: A Randomized‐Controlled Trial	*Health Education Journal*	82	2	169–183	10.1177/0017896922 1146429
K. Gur; S. Erol; N. Incir	2018	The Effectiveness of a Nail‐Biting Prevention Program Among Primary School Students	*Journal for Specialists in Pediatric Nursing: JSPN*	23	3	e12219	10.1111/jspn.12219
L. Henry; C. W. Smithson; L. M. Steurer; P. M. Ercole	2022	The Feasibility of a School Nurse–Led Mindfulness Program	*Journal of School Nursing*	38	6	519–525	10.1177/1059840521 1001833
H. Hiscock; J. Quach; K. Paton; R. Peat; L. Gold; S. Arnup; K.‐L. Sia; E. Nicolaou; M. A.‐H. Wake	2019	Impact of a Behavioral Sleep Intervention on New School Entrants' Social Emotional Functioning and Sleep: A Translational Randomized Trial	*Behavioral Sleep Medicine*	17	6	698–712	10.1080/15402002.2018.1469493
A. Holka	2014	Advocating for Students With Food Allergies	*Allergy and Asthma Today*	12	1	17–17	
A. Ilgaz	2022	Effect of Health Screening and School Nurse Interventions on Primary School Students' Knowledge, Behavior, and Status in Turkey: A Quasi‐Experimental Omaha System Study	*Journal of Pediatric Nursing*	62		e115‐e124	10.1016/j.pedn.2021.08.014
T. L. Jakubowski; E. M. Alexy	2014	Does School Scoliosis Screening Make the Grade?	*NASN School Nurse*	29	5	258–265	10.1177/1942602X1 4542131
S.‐J. Kim; H. Cho; S.‐S. Baek	2016	Effects of Healthy Life Practice Education on Reported Health Behaviors Among Fourth‐Grade Elementary School Students in South Korea	*Journal of School Nursing*	32	6	397–406	10.1177/1059840516 650261
M. Y. Kubik; J. A. Fulkerson; J. R. Sirard; A. Garwick; J. Temple; O. Gurvich; J. Lee; B. Dudovitz	2018	School‐Based Secondary Prevention of Overweight and Obesity Among 8‐ to 12‐Year Old Children: Design and Sample Characteristics of the SNAPSHOT Trial	*Contemporary Clinical Trials*	75	101,242,342	9–18	10.1016/j.cct.2018.10.011
M. Y. Kubik; J. Lee; J. A. Fulkerson; O. V. Gurvich; J. R. Sirard	2021	School‐Based Secondary Obesity Prevention for Eight‐ to Twelve‐Year‐Olds: Results from the Students, Nurses, and Parents Seeking Healthy Options Together Randomized Trial	*Childhood Obesity*	17	3	185–195	10.1089/chi.2020.0321
J. Lee; M. Y. Kubik	2015	Child's Weight Status and Parent's Response to a School‐Based Body Mass Index Screening and Parent Notification Program	*The Journal of School Nursing*	31	4	300–305	10.1177/105984051 4556181
Y. Li; S. Duffy; S. Wilks; R. Keel; R. Beswick; S. Dai	2023	Positive Predictive Value of Dual‐Modality Vision Screening in School Children 4–7 years of Age—A Retrospective Review in Queensland, Australia	*Journal of AAPOS: The official Publication of the American Association for Pediatric Ophthalmology and Strabismus*	27	1	22.e1‐22.e5	10.1016/j.jaapos.2022.11.009
M. J. Lineberry; M. J. Ickes	2015	The Role and Impact of Nurses in American Elementary Schools: A Systematic Review of the Research	*The Journal of School Nursing*	31	1	22–33	10.1177/105984051454 0940
D. R. Liptzin; M. C. Gleason; L. C. Cicutto; C. L. Cleveland; D. J. Shocks; M. K. White; A. V. Faino; S. J. Szefler	2016	Developing, Implementing, and Evaluating a School‐Centered Asthma Program: Step‐Up Asthma Program	*The Journal of Allergy And Clinical Immunology. In Practice*	4	5	972‐979.e1	10.1016/j.jaip.2016.04.016
C. M. Lovell	2018	5‐2‐1‐0 Activity and Nutrition Challenge for Elementary Students: New, Evidence‐Based, Promising	*The Journal of School Nursing*	34	2	98–107	10.1177/10598405 17689893
B. C. Mbakaya; R. L. T. Lee	2019	Experiences of Implementing Hand Hygiene for Malawian Schoolchildren: A Qualitative Study	*International Nursing Review*	66	4	553–562	10.1111/inr.12538
S. McClendon; M. B. Zeni	2020	Evaluation of Vision Referral Program With School‐Aged Children and Their Parents/Guardians	*Journal of School Nursing*	36	4	243–250	10.1177/1059840518 821427
C. F. Mickel; K. K. Shanovich; M. D. Evans; D. J. Jackson	2017	Evaluation of a School‐Based Asthma Education Protocol	*The Journal of School Nursing*	33	3	189–197	10.1177/1059840516 659912
M. Moberg	2023	School Nurses' Use of Motivational Interviewing as a Method for Health Promoting Conversations With Parents of Primary School Children	*Dissertation Abstracts International: Section B: The Sciences and Engineering*	84	6	Not Specified	
E. M. Moron; R. Singer	2023	An Interprofessional School‐Based Initiative to Increase Access to Oral Health Care in Underserved Florida Counties	*Journal of Public Health Dentistry*	83	2	155–160	10.1111/jphd.12562
R. Nash; K. Patterson; A. Flittner; S. Elmer; R. Osborne	2021	School‐Based Health Literacy Programs for Children (2–16 Years): An International Review	*The Journal of School Health*	91	8	632–649	10.1111/josh.13054
M. Otero‐Agra; L. Rey‐Fernández; D. Pacheco‐Rodríguez; F. Fernández‐Méndez; R. Barcala‐Furelos; R. Greif	2023	Pediatric Manikins and School Nurses as Basic Life Support Coordinators: A Useful Strategy for Schools?	*Health Education Journal*	82	1	3–16	10.1177/00178969221 133238
I. Puspitasari; S. Mulyono; D. N. Kusumawati	2019	The Effect of Interactive Education With 3‐Dimensional Puzzles on the Injury‐Prevention Behaviours of School‐Age Children	*Comprehensive Child and Adolescent Nursing*	42		173–178	10.1080/24694193.2019.1578438
M. Rabner; K. Bissett; S. B. Johnson; K. A. Connor	2020	A Risk Stratification Algorithm for Asthma Identification and Prioritization in a Low‐Income Urban School	*The Journal of School Health*	90	7	538–544	10.1111/josh.12903
P. A. Rademacher	2013	The Nurse in the School Health Office: Exploring Health Care in a Public School	*Dissertation Abstracts International Section A: Humanities and Social Sciences*	73	8	Not specified	
D. G. Ruggieri; S. B. Bass; M. Alhajji; T. F. Gordon	2020	Understanding Parents' Perceptions of School‐Based BMI Screening and BMI Report Cards Using Perceptual Mapping: Implications for School Nurses	*Journal of School Nursing*	36	2	144–156	10.1177/10598405 18789243
H. Ryu; Y. Lee	2015	The Effects of a School‐Based Atopy Care Program for School‐Aged Children	*Western Journal of Nursing Research*	37	8	1014–1032	10.1177/01939459 14539737
C. Schultz; A. E. Rosen	2022	School Gardens' Impact on Students' Health Outcomes in Low‐Income Midwest Schools	*Journal of School Nursing*	38	5	486–493	10.1177/10598405221 080970
E. Supriatun; A. Y. Nursasi; P. Fitriyani	2017	Enhancement of Pulmonary Tuberculosis Prevention Behavior With Role Play Among Elementary School Students	*Comprehensive Child and Adolescent Nursing*	40		78–87	10.1080/24694193.2017.1386974
P. G. Szilagyi; S. Schaffer; C. M. Rand; N. P. N. Goldstein; M. Younge; M. Mendoza; C. S. Albertin; C. Concannon; E. Graupman; A. D. Hightower; B.‐K. Yoo; S. G. Humiston	2019	Text Message Reminders for Child Influenza Vaccination in the Setting of School‐Located Influenza Vaccination: A Randomized Clinical Trial	*Clinical Pediatrics*	58	4	428–436	10.1177/00099228 18821878
J. Takaya; H. Higashino; R. Takaya; H. Sakaguchi; J. Tanoue; T. Higashide; H. Moriguchi; M. Nakao; S. Shigematsu	2022	Impact of Childhood Growth and Obesity Curves in School Health Examinations	*Pediatrics International: Official Journal of the Japan Pediatric Society*	64	1	e15182	10.1111/ped.15182
J. A. Tipton	2016	Reducing Sugar‐Sweetened Beverage Intake Among Students	*NASN School Nurse*	31	2	102–110	10.1177/1942602X15 578456
S. Tucker; L. M. Lanningham‐Foster	2015	Nurse‐Led School‐Based Child Obesity Prevention	*The Journal of School Nursing*	31	6	450–466	10.1177/1059840515 574002
V. Vejzovic; L. Carlson; L. Löfgren; A.‐C. Bramhagen	2022	Early Identification of Mental Illness in Primary School Pupils by School Nurses: A Qualitative Study	*SAGE Open Nursing*	8		1–7	10.1177/23779608221 081452
M. A. Willgerodt; G. M. Kieckhefer; T. M. Ward; M. J. Lentz	2014	Feasibility of Using Actigraphy and Motivational‐Based Interviewing to Improve Sleep Among School‐Age Children and Their Parents	*The Journal of School Nursing*	30	2	136–148	10.1177/105984051 3489711
D. Wilson; K. M. Sanchez; S. H. Blackwell; E. Weinstein; A. N. El Amin	2013	Implementing and Sustaining School‐Located Influenza Vaccination Programs: Perspectives From Five Diverse School Districts	*Journal of School Nursing*	29	4	303–314	10.1177/1059840513 486011
K. Wright; J. N. Giger; K. Norris; Z. Suro	2013	Impact of a Nurse‐Directed, Coordinated School Health Program to Enhance Physical Activity Behaviors and Reduce Body Mass Index Among Minority Children: A Parallel‐Group, Randomized Control Trial	*International Journal of Nursing Studies*	50	6	727–737	10.1016/j.ijnurstu.2012.09.004

## Appendix B Risk of Bias Assessment (see Tables B1, B2, B3, B4, B5)

**TABLE B1:** Risk of bias assessed using ROBINS‐I tool.

Study	ROBINS‐I tool—risk of bias domains							
	Bias due to confounding	Bias in selection of participants into the study	Bias in classification of interventions	Bias due to deviations from intended interventions	Bias due to missing data	Bias in measurement of outcomes	Bias in selection of the reported result	Overall judgment
Lovell 2017	Serious	Low	Low	Low	Serious	Serious	Moderate	Serious
Anderson et al. 2016	Low	Low	Low	Low	Low	Low	Low	Low
Cheng et al. 2021	Low	Low	Low	Low	Moderate	Low	Low	Moderate
Gür et al. 2017	Moderate	Low	Low	Low	Low	Serious	Low	Serious
Henry et al. 2022	Serious	Moderate	Low	Low	Low	Moderate	Low	Serious
Rabner et al. 2020	Serious	Low	Low	Low	Low	Low	Low	Serious
Li et al. 2022	Moderate	Low	Low	Low	Moderate	Moderate	Low	Moderate
Moron 2023	Moderate	Low	Low	Low	Low	Low	Low	Moderate
Tucker et al. 2015	Serious	Low	Low	Low	Moderate	Low	Low	Serious

*Note*: “X” means that the respective column applies (the answer to the question on the checklist is “Yes” or “No”).

**TABLE B2:** Risk of bias assessed using RoB 2 tool.

Study	RoB 2 tool—risk of bias domains					
	Bias arising from the randomization process	Bias due to deviations from intended interventions	Bias due to missing outcome data	Bias in measurement of the outcome	Bias in selection of the reported result	Overall judgment
Szilagyi et al. 2019	Low	Some concerns	Low	Low	Low	Some concerns
Çövener‐Özcelik et al. 2017	Low	High	Some concerns	Low	Low	High
Ergün et al. 2012	Low	High	Low	Low	Low	High
Ergun et al. 2013	Low	Some concerns	Low	Some concerns	Low	Some concerns
Goodarzi et al. 2020	Low	High	Low	Some concerns	Low	High
Güler et al. 2023	Low	High	Low	Low	Low	High
Kim et al. 2016	Low	Some concerns	Low	Some concerns	Low	Some concerns

*Note*: “X” means that the respective column applies (the answer to the question on the checklist is “Yes” or “No”).

**TABLE B3:** Risk of bias assessed using AMSTAR 2 tool.

Checklist for qualitative research	AMSTAR 2 in Lineberry and Ickes 2015		
	Yes	Partial yes	No
Did the research questions and inclusion criteria for the review include the components of PICO?			X
Did the report of the review contain an explicit statement that the review methods were established prior to the conduct of the review and did the report justify any significant deviations from the protocol?		X	
Did the review authors explain their selection of the study designs for inclusion in the review?			X
Did the review authors use a comprehensive literature search strategy?		X	
Did the review authors perform study selection in duplicate?			X
Did the review authors perform data extraction in duplicate?			X
Did the review authors provide a list of excluded studies and justify the exclusions?			X
Did the review authors describe the included studies in adequate detail?		X	
Did the review authors use a satisfactory technique for assessing the risk of bias (RoB) in individual studies that were included in the review?			X
Did the review authors report on the source of funding for the studies included in the review			X
If meta‐analysis was performed did the review authors use appropriate methods for statistical combination of results?			No meta‐analysis conducted
If meta‐analysis was performed, did the review authors assess the potential impact of RoB in individual studies on the results of the meta‐analysis or other evidence synthesis?			No meta‐analysis conducted
Did the review authors account for RoB in individual studies when interpreting/discussing the results of the review?			X
Did the review authors provide a satisfactory explanation for, and discussion of, any heterogeneity observed in the results of the review?			X
If they performed quantitative synthesis did the review authors carry out an adequate investigation of publication bias (small study bias) and discuss its likely impact on the results of the review?			No meta‐analysis conducted
Did the review authors report any potential sources of conflict of interest, including any funding they received for conducting the review?			X
Overall judgment/rating overall confidence in the results of the review	Critically low^a^		

*Note*: AMSTAR 2 is not intended to generate an overall score.

^a^ More than one critical flaw with or without noncritical weaknesses: The review has more than one critical flaw and should not be relied on to provide an accurate and comprehensive summary of the available studies. “X” means that the respective column applies (the answer to the question on the checklist is “Yes” or “No”).

**TABLE B4:** Risk of bias assessed using JsBI qualitative research.

Checklist for qualitative research	JBI qualitative research in Mbakaya and Lee 2019			
	Yes	No	Not clear	Not applicable
Is there congruity between the stated philosophical perspective and the research methodology?	X			
Is there congruity between the research methodology and the research question or objectives?	X			
Is there congruity between the research methodology and the methods used to collect data?	X			
Is there congruity between the research methodology and the representation and analysis of data?	X			
Is there congruity between the research methodology and the interpretation of results?	X			
Is there a statement locating the researcher culturally or theoretically?			X	
Is the influence of the researcher on the research, and vice‐versa, addressed?			X	
Are participants, and their voices, adequately represented?	X			
Is the research ethical according to current criteria or, for recent studies, and is there evidence of ethical approval by an appropriate body?	X			
Do the conclusions drawn in the research report flow from the analysis, or interpretation, of the data?	X			
Overall judgment	Some concerns			

**TABLE B5:** Risk of bias assessed using JBI narrative.

Critical appraisal checklist for textual evidence	JBI narrative in Tipton 2015			
	Yes	No	Not clear	Not applicable
Is the generator of the narrative a credible or appropriate source?	X			
Is the relationship between the text and its context explained? (where, when, who with, how)	X			
Does the narrative present the events using a logical sequence so the reader or listener can understand how it unfolds?	X			
Do you, as reader or listener of the narrative, arrive at similar conclusions to those drawn by the narrator?	X			
Do the conclusions flow from the narrative account?	X			
Do you consider this account to be a narrative?	X			
Overall judgment	Low			

## Appendix C Search Syntax (See Tables C1, C2, C3, C4, C5, C6, C7)

**TABLE C1:** Medline via OVID.

Subject	Syntax	Results
Elementary school	((((elementary OR primary) adj3 school*) OR grade school* OR grundschule?).ti,ab,kw.) AND	
School nurse	(((school* adj3 nurs*) not (school? of nursing OR nursery school? OR nursing school?)).ti,ab,kw. OR	
	School Nursing/) AND	
Health promotion	(((health adj3 promot*) OR prevent*).ti,ab,kw. OR	
	(health adj3 (service? OR intervent* OR program*)).ti,ab,kw. OR	
	((health adj3 education) OR counsel* OR screening OR health literacy).ti,ab,kw. OR	
	(Gesundheitsforder* OR Praventi*).ti,ab,kw. OR	
	exp Health Promotion/ OR	
	exp Primary Prevention/)	
Restriction	limit to yr=“2013‐Current”	167

**TABLE C2:** PsycINFO via OVID.

Subject	Syntax	Results
Elementary school	((((elementary OR primary) adj3 school*) OR "grade school*" OR grundschule?).mp. OR	
	elementary schools/) AND	
School nurse	((school* adj3 nurs* not ("school? of nursing" OR "nursery school?" OR "nursing school?")).mp. OR	
	school nurses/) AND	
Health promotion	(((health adj3 (promot* OR service? OR intervent* OR program* OR education)) OR prevent* OR counsel* OR screening OR health literacy).mp. OR	
	Health Promotion/ OR	
	Prevention/ OR	
	Preventive Health Services/ OR	
	exp School Based Intervention/ OR	
	Health Education/ OR	
	exp Health Literacy/)	
Restriction	limit to yr=“2013‐Current”	80

**TABLE C3:** CINAHL via EBSCO.

Subject	Syntax	Results
Elementary school	(((elementary OR primary OR grade) school*) OR grundschule?) OR	
	(MH "Schools, elementary")) AND	
School nurse	(("school nurs*") OR	
	(MH "School Nurses") OR	
	(MH "School Nursing")) AND	
Health promotion	(("health promot*" OR "health service?" OR "health intervention?" OR "health program?" OR "health education" OR preventi* OR counsel#ing OR screening OR "health literacy" OR Gesundheitsförderung OR Prävention) OR	
	(MH "School Health Services+") OR	
	(MH "Student Health Services+") OR	
	(MH "Health Promotion+" OR MH "Preventive Health Care+") OR	
	(MH "Health Education+" OR MH "Dental Health Education") OR	
	(MH "Health Literacy") OR	
	(MH "Health Screening+"))	
Restriction	limit to yr=“2013‐Current”	299

**TABLE C4:** Education Resources Information Center by US Department of Education via ProQuest.

Subject	Syntax	Results
Elementary school	(ABSTRACT,TITLE("elementary school" OR "elementary schooling" OR "elementary schools" OR "primary school" OR "primary schooling" OR "primary schools" OR "grade school" OR "grade schooling" OR "grade schools" or grundschule or grundschulen) OR	
	MAINSUBJECT.EXACT("Elementary School Students") OR	
	MAINSUBJECT.EXACT("Elementary Schools")) AND	
School Nurse	(ABSTRACT,TITLE("school nurse" OR "school nursing" OR "school nurses") OR	
	MAINSUBJECT.EXACT("School Nurses")) AND	
Health Promotion	(ABSTRACT,TITLE("health promoting" OR "health promotion" OR "health service" OR "health services" OR "health intervention" OR "health interventions" OR "health program" OR "health programs" OR "health programme" OR "health programmes" OR "health education" OR preventing OR prevention OR counseling OR counselling OR screening OR "health literacy" OR Gesundheitsforderung OR Pravention) OR	
	MAINSUBJECT.EXACT("Health Promotion") OR	
	MAINSUBJECT.EXACT("Prevention") OR	
	MAINSUBJECT.EXACT("School Health Services") OR	
	MAINSUBJECT.EXACT.EXPLODE("Health Education") OR	
	MAINSUBJECT.EXACT("screening tests"))	
Restriction	pubyearmin:2013	52

**TABLE C5:** Scopus.

Subject	Syntax	Results
Elementary school	TITLE‐ABS‐KEY ("elementary school*" OR "primary school*" OR "grade school*" OR grundschule?) AND	
School nurse	TITLE‐ABS‐KEY ("school nurs*") AND	
Health promotion	TITLE‐ABS‐KEY ( "health promot*" OR "health service?" OR "health intervention?" OR "health program?" OR "health education" OR preventi* OR counsel#ing OR screening OR "health literacy" OR Gesundheitsforderung OR Pravention) AND	
Restriction	PUBYEAR>2012 AND PUBYEAR<2024	115

**TABLE C6:** Applied Social Sciences Index and Abstracts via ProQuest.

Subject	Syntax	Results
Elementary school	ABSTRACT,TITLE(((elementary or primary) adj3 school*) or grade school* OR grundschule?)) AND	
	(MAINSUBJECT.EXACT("Elementary Schools") OR	
School nurse	(ABSTRACT,TITLE(school* adj3 nurs* not (school? of nursing or nursery school? or nursing school?)) OR	
	MAINSUBJECT.EXACT("School Nurses")) AND	
Health promotion	(ABSTRACT,TITLE(health adj3 (promot* OR service? OR intervention? OR program* OR education) OR prevent* OR counsel* OR screening OR "health literacy" OR Gesundheitsforderung OR Pravention) OR	
	MAINSUBJECT.EXACT("Health promotion") OR	
	MAINSUBJECT.EXACT("Mental health promotion") OR	
	MAINSUBJECT.EXACT("Preventive medicine") OR	
	MAINSUBJECT.EXACT("Disease prevention"))	
Restriction	Filter “2013‐2023”	29

**TABLE C7:** Search Conducted on 12.09.2023.

Database	Results
Medline via Ovid	167
PsycINFO via Ovid	80
CINAHL via EBSCO	299
Education Resources Information Center by US Department of Education via ProQuest	52
Scopus	115
Applied social sciences index and abstracts via ProQuest	29
Total results imported to Rayyan	742
Duplicates detected by Rayyan	419
Results for screening	486
